# Trends in COVID-19 Cases, Emergency Department Visits, and Hospital Admissions Among Children and Adolescents Aged 0–17 Years — United States, August 2020–August 2021

**DOI:** 10.15585/mmwr.mm7036e1

**Published:** 2021-09-10

**Authors:** David A. Siegel, Hannah E. Reses, Andrea J. Cool, Craig N. Shapiro, Joy Hsu, Tegan K. Boehmer, Cheryl R. Cornwell, Elizabeth B. Gray, S. Jane Henley, Kimberly Lochner, Amitabh B. Suthar, B. Casey Lyons, Linda Mattocks, Kathleen Hartnett, Jennifer Adjemian, Katharina L. van Santen, Michael Sheppard, Karl A. Soetebier, MAPW, Pamela Logan, Michael Martin, Osatohamwen Idubor, Pavithra Natarajan, Kanta Sircar, Eghosa Oyegun, Joyce Dalton, Cria G. Perrine, Georgina Peacock, Beth Schweitzer, Sapna Bamrah Morris, Elliot Raizes

**Affiliations:** ^1^CDC COVID-19 Response Team; ^2^Booz Allen Hamilton, McLean, Virginia; ^3^Oak Ridge Institute for Science and Education, Oak Ridge, Tennessee; ^4^ICF International, Fairfax, Virginia; ^5^Tanaq Support Services, LLC, Anchorage, Alaska; ^6^Division of Human Development and Disability, National Center on Birth Defects and Developmental Disabilities, CDC.

Although COVID-19 generally results in milder disease in children and adolescents than in adults, severe illness from COVID-19 can occur in children and adolescents and might require hospitalization and intensive care unit (ICU) support (*1*–*3*). It is not known whether the B.1.617.2 (Delta) variant,* which has been the predominant variant of SARS-CoV-2 (the virus that causes COVID-19) in the United States since late June 2021,^†^ causes different clinical outcomes in children and adolescents compared with variants that circulated earlier. To assess trends among children and adolescents, CDC analyzed new COVID-19 cases, emergency department (ED) visits with a COVID-19 diagnosis code, and hospital admissions of patients with confirmed COVID-19 among persons aged 0–17 years during August 1, 2020–August 27, 2021. Since July 2021, after Delta had become the predominant circulating variant, the rate of new COVID-19 cases and COVID-19–related ED visits increased for persons aged 0–4, 5–11, and 12–17 years, and hospital admissions of patients with confirmed COVID-19 increased for persons aged 0–17 years. Among persons aged 0–17 years during the most recent 2-week period (August 14–27, 2021), COVID-19–related ED visits and hospital admissions in the states with the lowest vaccination coverage were 3.4 and 3.7 times that in the states with the highest vaccination coverage, respectively. At selected hospitals, the proportion of COVID-19 patients aged 0–17 years who were admitted to an ICU ranged from 10% to 25% during August 2020–June 2021 and was 20% and 18% during July and August 2021, respectively. Broad, community-wide vaccination of all eligible persons is a critical component of mitigation strategies to protect pediatric populations from SARS-CoV-2 infection and severe COVID-19 illness.

CDC analyzed COVID-19 cases, ED visits with a COVID-19 diagnosis code, and hospital admissions of patients with laboratory-confirmed COVID-19 among persons aged 0–17 years during August 1, 2020–August 27, 2021. Daily COVID-19 case data were obtained from CDC’s case-based surveillance system.^§^ Daily ED visits were obtained from the National Syndromic Surveillance Program^¶^ and were stratified into three age groups: 0–4, 5–11, and 12–17 years. Daily hospital admission data were obtained from the U.S. Department of Health and Human Services (HHS) Unified Hospital Data Surveillance System.** The number of daily cases, ED visits, and hospital admissions were averaged over a 7-day period to obtain a 7-day average. The state-specific percentage of the population aged ≥12 years who had completed the COVID-19 vaccination series as of July 31, 2021, was used to group states into vaccination coverage quartiles.^††^ Results were also examined by HHS Region. U.S. Census Bureau midyear 2019 population estimates^§§^ were used to calculate vaccination coverage and cases and hospital admissions per 100,000 persons. COVID-19–associated ED visits were assessed as a percentage of all ED visits. To assess differences in COVID-19 outcomes by vaccination coverage quartile, ratios for ED visits and rate ratios for hospital admissions during the 2-week period August 14–27, 2021, along with corresponding 95% confidence intervals (CIs), were calculated. R (Version 4.1.0; R Foundation) was used for calculations.

The BD Insights Research Database was used to describe indicators of severe disease among pediatric patients hospitalized with laboratory-confirmed COVID-19.^¶¶^ CDC analyzed the monthly percentage of hospitalizations resulting in ICU admission and in invasive mechanical ventilation, and median length of hospital stay during August 1, 2020–August 21, 2021. These analyses were conducted using SAS (version 9.4; SAS Institute).*** This activity was reviewed by CDC and was conducted consistent with applicable federal law and CDC policy.^†††^

COVID-19 incidence among persons aged 0–4, 5–11, and 12–17 years during August 2020–August 2021 peaked in January 2021 at 21.2, 30.1, and 51.7 cases per 100,000 persons, respectively (Figure 1). Incidence declined in June 2021 to a low of 1.7, 1.9, and 2.9, respectively, across the three age groups; however, incidence in August 2021 among the three age groups reached 16.2, 28.5, and 32.7 per 100,000 persons, respectively. 

**FIGURE 1:**
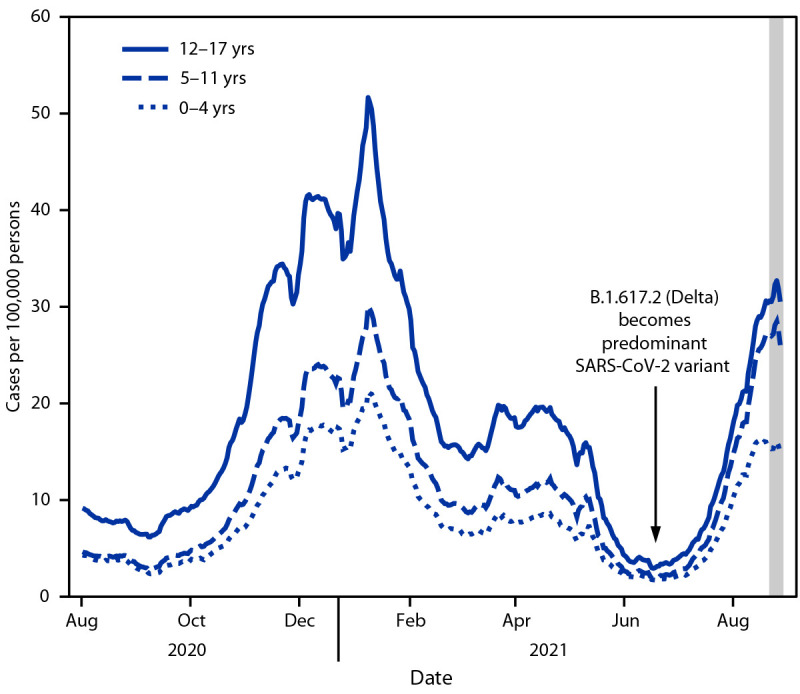
Average daily COVID-19 case incidence* among persons aged 0–17 years, by age group — United States, August 1, 2020–August 27, 2021 **Source:** CDC’s case-based COVID-19 surveillance system, accessed August 30, 2021. https://www.cdc.gov/nndss/action/covid-19-response.html * Incidence calculated as daily cases averaged over a 7-day period to obtain a 7-day moving daily average per 100,000 persons using 2019 U.S. Census population as denominators (three age groups: 0–4, 5–11, and 12–17 years). Delta became the predominant SARS-CoV-2 variant in the United States in late June 2021, accounting for 63% of new COVID-19 cases the week ending June 26, 2021 (https://covid.cdc.gov/covid-data-tracker/#variant-proportions). Because of potential reporting delays, data reported in the most recent 7 days (as represented by the shaded bar) should be interpreted with caution.

Overall, COVID-19 ED visits and hospital admissions increased since June 2021 among states in all vaccination coverage quartiles (Supplementary Figure, https://stacks.cdc.gov/view/cdc/109403). The percent of COVID-19 ED visits in August 2021 in the quartile of states with the lowest vaccination coverage was 3.4 times that in the quartile of states with the highest vaccination coverage (Table). The rate (per 100,000 persons) of COVID-19 admissions in August 2021 in the quartile of states with the lowest vaccination coverage was 3.7 times that in the quartile of states with the highest vaccination coverage.

The lowest vaccination coverage among persons aged ≥12 years (49.9%), highest percentage of COVID-19–associated ED visits (8.32), and highest COVID-19 hospital admission rates (0.84) were observed in HHS Region 4.^§§§^ In contrast, the highest vaccination coverage (72.2%), lowest COVID-19 incidence (13.3), and lowest rate of hospital admission (0.12) among persons aged 0–17 years were observed in HHS Region 1 (Supplementary Table, https://stacks.cdc.gov/view/cdc/109402).

In the BD Insights Research Database, 1,790 COVID-19 hospitalizations occurred among persons aged 0–17 years during August 1, 2020–August 21, 2021. Median length of stay ranged from 2 to 3 days during the entire period. The percentage of hospitalizations resulting in an ICU admission ranged from 10% to 25% during August 2020–June 2021; percentages were 20% and 18% in July and August 2021, respectively (Figure 2). The percentage of hospitalizations resulting in invasive mechanical ventilation ranged from 0% to 3% and was highest in October 2020; percentages in July and August 2021 were 2% and <1%, respectively. A total of eight in-hospital COVID-19–related deaths in persons aged 0–17 years occurred during August 2020–August 2021 (0.4% of hospitalized patients). Among 63 patients aged 0–17 years admitted to an ICU in July and August 2021, 17 (27%) were aged 0–4 years, 17 (27%) were 5–11 years, and 29 (46%) were 12–17 years.

**FIGURE 2:**
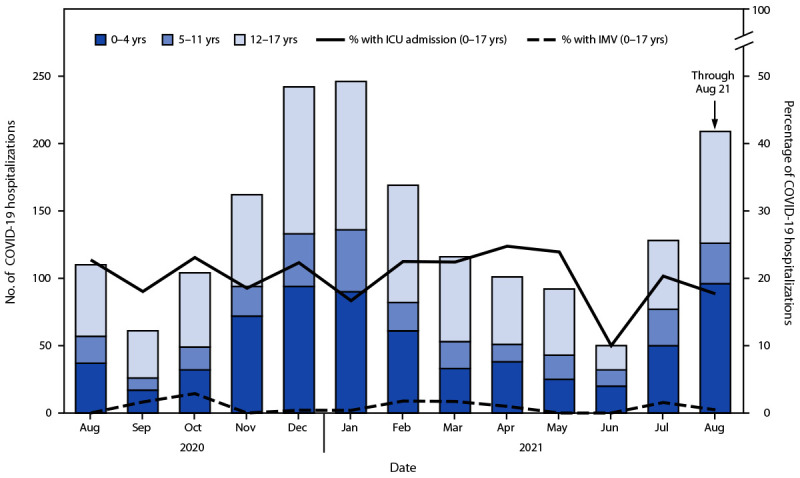
Number and percentage of COVID-19 hospitalizations resulting in intensive care unit admission or invasive mechanical ventilation among persons aged 0–17 years, by age group — United States, August 1, 2020–August 21, 2021 **Source:** BD Insights Research Database. **Abbreviations:** ICU = intensive care unit; IMV = invasive mechanical ventilation.

## Discussion

Among U.S. children and adolescents aged 0–17 years, COVID-19 cases and associated ED visits and hospital admissions increased during June 2021–August 2021. During a 2-week period in August 2021, COVID-19–associated ED visits and hospital admissions for children and adolescents with confirmed COVID-19 were highest in states with lowest vaccination coverage, particularly states in the South, whereas in the states with the highest coverage, COVID-19 ED visits and the rate of hospital admissions among children and adolescents were lowest. These findings underscore the importance of community vaccination, in coordination with testing strategies and other prevention measures, to protect children from SARS-CoV-2 infection and severe COVID-19.

Children and adolescents can experience severe acute COVID-19, which might require mechanical ventilation, or result in other complications, such as multisystem inflammatory syndrome in children (MIS-C) (*2*)^¶¶¶^ and persistent symptoms from COVID-19 (*4*). Pediatric patients who have underlying medical conditions might be at risk for more severe disease (*3*). The increases in COVID-19 hospital admissions found in this study occurred for all assessed pediatric age groups during July–August 2021, with most admissions among patients aged ≤4 or 12–17 years. This bimodal age distribution is consistent with other published data (*5*).

Increases in COVID-19 ED visits and hospital admissions were observed in all four state COVID-19 vaccination coverage quartiles during June–August 2021. Although some data suggest that persons infected with the Delta SARS-CoV-2 variant might be at higher risk for hospitalization (*6*), it is not clear whether the Delta variant causes more severe illness in adult or pediatric populations. Although it is possible that increases in COVID-19–related ED visits and hospital admissions for pediatric patients with confirmed COVID-19 could be related to increased severity of disease for the Delta variant compared with severity for earlier circulating variants, increases in ED visits and hospitalizations could be related to other factors such as increased transmission (*6*).****

Pediatric ED visits and hospital admissions were higher in August 2021 in states with the lowest vaccination coverage among persons aged ≥12 years. Although the SARS-CoV-2 Delta variant is highly transmissible, only a modest decrease in vaccine effectiveness against infection with the Delta variant has been reported (*7*); therefore, transmission might be a major factor driving increases in ED visits and hospital admissions. However, beyond community vaccination coverage, other factors driving regional variation might include differences in implementation of other prevention measures, including masking, physical distancing, and kindergarten through grade 12 (K–12) school opening policies (*8*).

This analysis found that the percentage of COVID-19 hospitalizations resulting in ICU admission has remained near 20% since Delta became the predominant SARS-CoV-2 variant. A study of children and adolescents hospitalized for COVID-19 during March 2020–July 2021 found that the proportion of those patients admitted to an ICU during the pre-Delta period (March 1, 2020–June 19, 2021) and the Delta-predominant period (June 20–July 31, 2021) did not differ (26.5% and 23.2%, respectively) (*9*). This same study found a median length of stay of 3 days during the pre-Delta period and 2 days during the Delta-predominant period among hospitalized patients aged 0–17 years with COVID-19 (*9*).

The findings in this report are subject to at least seven limitations. First, data sets used to quantify COVID-19 cases, ED visits, and hospital admissions are subject to reporting inconsistencies. Second, testing rates for SARS-CoV-2 infection in persons aged 0–17 years are lower than they are in older age groups^††††^; therefore, pediatric case rates are likely underreported. Third, ED visits and hospital admissions were not characterized by reason for visit or admission and might include cases of MIS-C or asymptomatic SARS-CoV-2 infection. Fourth, admissions in the Unified Hospital Data Surveillance System could not be stratified by age and were only counted if the patient was “admitted to a pediatric bed.”^§§§§^ Fifth, because the BD Insights Research Database represents three children’s hospitals and the remainder of patients were mostly from community hospitals, patients with severe COVID-19 might be under- or overrepresented, which might account for some differences compared with past studies. Sixth, patients are added to this database on hospital discharge; therefore, recent data do not include patients currently admitted. Finally, geographic representativeness varies across data sources.

Continued assessment of trends in ED visits and admissions, including evaluation of reason for seeking medical care, could help guide public health practice, including planning for any decreases in pediatric care capacity. Evaluating more specific measures of severity (e.g., hypoxia and duration of mechanical ventilation), potential co-infection (e.g., respiratory syncytial virus), vaccination status, and underlying medical conditions might help determine whether children and adolescents infected with the Delta variant have more severe disease than do those infected with other variants. As schools resume in-person activities, CDC recommends multiple prevention measures in early child care and education programs and K–12 schools,^¶¶¶¶^ such as masking for students and staff members and maintaining adequate ventilation to reduce transmission of SARS-CoV-2. Vaccination of eligible persons against COVID-19, especially those in close contact with children aged <12 years who are not yet eligible for vaccination, is anticipated to protect students, teachers, staff members, visitors, and other household members. Community vaccination, in coordination with testing strategies and other prevention measures, are critical to protecting pediatric populations from SARS-CoV-2 infection and severe COVID-19.

**Summary**

**What is already know about this topic?**

Severe illness from COVID-19 can and does occur in children and adolescents.

**What is added by this report?**

COVID-19 cases, emergency department visits, and hospital admissions increased from June to August 2021 among persons aged 0-17 years. Emergency department visits and hospital admissions in a 2-week period in August 2021 were higher in states with lower population vaccination coverage and lower in states with higher vaccination coverage.

**What are the implications for public health?**

Community vaccination, in coordination with testing strategies and other prevention measures, is critical to protecting pediatric populations from SARS-CoV-2 infection and severe COVID-19.

**TABLE:** Ratio of percentage of COVID-19–associated emergency department visits among all emergency department visits and rate ratio of COVID-19 hospital admissions* (compared with highest vaccination coverage quartile states) among persons aged 0–17 years, by quartile of states grouped by vaccination coverage and age group — United States, August 14, 2021–August 27, 2021^†^

State vaccination coverage quartile^§^	Ratio (95% CI)				
	ED visits				Hospital admissions
	0–17 yrs	0–4 yrs	5–11 yrs	12–17 yrs	0–17 yrs
Highest^¶^	Ref	Ref	Ref	Ref	Ref
Second highest**	0.99 (0.94–1.05)	1.02 (0.93–1.12)	0.99 (0.90–1.10)	0.96 (0.89–1.05)	1.40 (0.87–2.25)
Second lowest^††^	2.65 (2.55–2.76)	2.31 (2.15–2.47)	2.64 (2.44–2.84)	2.84 (2.67–3.03)	3.46 (2.26–5.28)
Lowest^§§^	3.38 (3.24–3.52)	2.61 (2.42–2.82)	3.34 (3.08–3.61)	3.76 (3.52–4.02)	3.70 (2.32–5.90)

**Sources**: COVID-19 Vaccination Trends in the United States (https://data.cdc.gov/Vaccinations/COVID-19-Vaccination-Trends-in-the-United-States-N/rh2h-3yt2) and National Syndromic Surveillance Program, U.S. Department of Health and Human Services Unified Hospital Data Surveillance System, accessed August 30, 2021.

**Abbreviations:** ED = emergency department; Ref = referent group.

* The hospital admission incidence rates that were used to calculate the rate ratios are 14-day average daily confirmed COVID-19 pediatric admissions per 100,000 persons aged 0–17 years. Rate in the lowest vaccination coverage quartile excludes data from Georgia because of a data quality issue.

^†^ ED visit data are from the National Syndromic Surveillance Program (NSSP). Data are limited to ED visits with a discharge diagnosis. Data from Hawaii and Ohio are not included. Fewer than 50% of facilities in California, Iowa, Minnesota, South Dakota, and Wyoming report to NSSP. In HHS Region 7, fewer than 50% of all ED visits have a discharge diagnosis.

^§^ Vaccine coverage data are for the population aged ≥12 years who completed COVID-19 vaccination series as of July 31, 2021. Idaho provides vaccine data only for vaccine recipients who are aged ≥18 years.

^¶^ Highest vaccination states: Connecticut, Maine, Maryland, Massachusetts, New Hampshire, New Jersey, New Mexico, New York, Oregon, Rhode Island, Vermont, Virginia, and Washington (>63.45%).

** Second highest vaccination states: California, Colorado, District of Columbia, Delaware, Hawaii, Illinois, Iowa, Michigan, Minnesota, Nebraska, Pennsylvania, and Wisconsin (>56.30% and ≤63.45%).

^††^ Second lowest vaccination states: Alaska, Arizona, Florida, Indiana, Kansas, Kentucky, Montana, Nevada, North Carolina, Ohio, South Dakota, Texas, and Utah (>49.75% and ≤56.30%).

^§§^ Lowest vaccination states: Alabama, Arkansas, Georgia, Idaho, Louisiana, Mississippi, Missouri, North Dakota, Oklahoma, South Carolina, Tennessee, West Virginia, and Wyoming (≤49.75%).

## Footnotes

* https://www.cdc.gov/coronavirus/2019-ncov/variants/variant-info.html#Concern

§ CDC official counts of COVID-19 cases and deaths, released daily (https://covid.cdc.gov/covid-data-tracker), are aggregate counts from reporting jurisdictions. Some jurisdictions electronically submit standardized information for individual cases of COVID-19 to CDC via a case report form developed for the CDC COVID-19 response (https://www.cdc.gov/coronavirus/2019-ncov/php/reporting-pui.html) or via the CDC National Notifiable Diseases Surveillance System (https://www.cdc.gov/nndss/action/covid-19-response.html). Individual-level case report data were available for approximately 80% of the aggregate number of reported cases. A COVID-19 case is defined by detection of SARS-CoV-2 RNA or antigen in a respiratory specimen collected from a person (confirmed or probable case) according to the Council of State and Territorial Epidemiologists’ Update to the standardized surveillance case definition and national notification for COVID-19 (21-ID-01): https://cdn.ymaws.com/www.cste.org/resource/resmgr/ps/ps2021/21-ID-01_COVID-19.pdf.

¶ The National Syndromic Surveillance Program collects electronic health data, including ED visits with COVID-19 diagnoses, from a subset of hospitals in 49 states and the District of Columbia (71% of nonfederal EDs in the United States). ED visits for COVID-19 are defined as ED visits with any of the following: *International Classification of Diseases, Tenth Revision* codes U07.1 or J12.82 or Systematized Nomenclature of Medicine codes 840539006, 840544004, or 840533007. https://www.cdc.gov/nssp/overview.html

** The HHS Unified Hospital Data Surveillance System includes data from all U.S. hospitals registered with the Centers for Medicare & Medicaid Services (CMS) as of June 1, 2020, and from hospitals not CMS–registered but reporting COVID-19 data through this system since July 1, 2020. Data, including counts of new hospital admissions of patients with confirmed COVID-19 by age group, are reported to HHS either directly from facilities or via a state health department submission; on August 27, 2021, 97% of hospitals reported data. This analysis includes children’s, short-term acute care, long-term acute care, critical access, Veterans Administration, Defense Health Agency, and Indian Health Services hospitals and excludes psychiatric, rehabilitation, and religious nonmedical hospitals. Reporting guidelines are published in the HHS COVID-19 Guidance for Hospital Reporting and FAQs document (https://www.hhs.gov/sites/default/files/covid-19-faqs-hospitals-hospital-laboratory-acute-care-facility-data-reporting.pdf). The hospital admissions rate in the lowest vaccination coverage quartile states excludes data from Georgia because of a data quality issue.

†† Obtained from COVID-19 Data Tracker (https://covid.cdc.gov/covid-data-tracker/#vaccinations_vacc-total-admin-rate-total). Persons aged 12–17 years became eligible for vaccination on May 10, 2021. Age 12 years is the current lowest age of eligibility for COVID-19 vaccination. https://www.fda.gov/news-events/press-announcements/coronavirus-covid-19-update-fda-authorizes-pfizer-biontech-covid-19-vaccine-emergency-use

§§ Population estimates as of July 1, 2020. https://www.census.gov/data/tables/time-series/demo/popest/2010s-national-detail.html

¶¶ BD Insights Research Database is a large U.S. hospital-based database that includes inpatient data from approximately 260 hospitals in rural and urban areas. Of these, 140 hospitals had COVID-19 hospitalizations among patients aged 0–17 years during the study period; by U.S. Census Region, most hospitals were in the South (52%), followed by the Midwest (18%), West (16%), and Northeast (14%). COVID-19 hospitalizations were identified by positive results on SARS-CoV-2 reverse transcription–polymerase chain reaction or antigen testing. Data are updated weekly; release date August 25, 2021, access date August 25, 2021. The database includes three children’s hospitals; the remainder of patients were mostly admitted to community hospitals.

*** Statistical testing and CIs were not used for the BD Research Insights Database because of its small size and design (i.e., census of patient data from select hospitals); the convenience sample does not allow for the use of weights to adjust for complex sampling.

††† 45 C.F.R. part 46; 21 C.F.R. part 56; 42 U.S.C. Sect. 241(d); 5 U.S.C. Sect. 552a; 44 U.S.C. Sect. 3501 et seq.

§§§ HHS Region 4 includes Alabama, Florida, Georgia, Kentucky, Mississippi, North Carolina, South Carolina, and Tennessee. Hospital admissions data in this report do not include Georgia.

¶¶¶ Accessed September 1, 2021. https://www.cdc.gov/mis/index.html

**** Accessed September 1, 2021. https://www.cdc.gov/coronavirus/2019-ncov/variants/delta-variant.html

†††† COVID-19 electronic laboratory reporting data set, accessed August 29, 2021.

§§§§
https://www.hhs.gov/sites/default/files/covid-19-faqs-hospitals-hospital-laboratory-acute-care-facility-data-reporting.pdf

¶¶¶¶
https://www.cdc.gov/coronavirus/2019-ncov/community/schools-childcare/k-12-guidance.html
