# *Erratum:* Vol. 70, No. 36

**DOI:** 10.15585/mmwr.mm7038a5

**Published:** 2021-09-24

In the report “Trends in COVID-19 Cases, Emergency Department Visits, and Hospital Admissions Among Children and Adolescents Aged 0–17 years — United States, August 2020–August 2021,” on page 1252, in the Table, the second footnote should have read, “^†^ ED visit data are from the National Syndromic Surveillance Program (NSSP). Data are limited to ED visits with a discharge diagnosis. Data from Hawaii and Ohio are not included. Fewer than 50% of facilities in California, Iowa, Minnesota, and **Oklahoma** report to NSSP. In HHS Region 7, fewer than 50% of all ED visits have a discharge diagnosis.” On page 1253, the title for Figure 2 should have read, “Number **of COVID-19 hospitalizations** and percentage of COVID-19 hospitalizations resulting in intensive care unit admission or invasive mechanical ventilation among persons aged 0–17 years, by age group — United States, August 1, 2020–August 21, 2021.”

